# Supplementary material for: Imidazoles and Quaternary Ammonium Compounds as Effective Therapies against (Multidrug-Resistant) Bacterial Wound Infections
Source: Antibiotics (Basel). 2024 Oct 10;13(10):949. doi: 10.3390/antibiotics13100949 (PMC11505196; doi:10.3390/antibiotics13100949)
Supplement: Supplementary file 1 [file antibiotics-13-00949-s001.zip › antibiotics-3240947-supplementary.pdf]

Supplementary data

Table S1: Overview of the used concentration ranges (in µM) for the checkerboard assays. The replicate number with its corresponding concentration range is specified per bacterium and tested compound.

Bacterial strain	Replicate	Compound	Concentration range (Max-Min µM)	
A. baumannii RD5SR3	1	Miconazole	1000	2.0
		Domiphen bromide	100	3.1
	2-3	Miconazole	250	0.48
		Domiphen bromide	25	0.78
P. aeruginosa PA14	1	Miconazole	1000	2.0
		Domiphen bromide	100	3.1
	2-3	Miconazole	250	0.48
		Domiphen bromide	100	3.1
S. aureus SH1000	1	Miconazole	250	0.48
		Domiphen bromide	100	3.1
	2	Miconazole	1000	2.0
		Domiphen Bromide	100	3.1
	3-4	Miconazole	250	0.48
		Domiphen bromide	25	0.78
	1-2	Clotrimazole	250	0.48
		Domiphen bromide	100	3.1
	3-4,6	Clotrimazole	250	0.48
		Domiphen bromide	25	0.78
	5	Clotrimazole	350	0.68
		Domiphen bromide	100	3.1
	1-2	Ketoconazole	250	0.48
		Domiphen bromide	100	3.1
	3-5	Ketoconazole	250	0.48
		Domiphen bromide	25	0.78
	1	Voriconazole	1000	2.0
		Domiphen bromide	100	3.1
	2-3	Voriconazole	250	0.49
		Domiphen bromide	100	3.1
	1-3	Fluconazole	1000	2.0
		Domiphen bromide	100	3.12
	1-4	Miconazole	250	0.49
		Benzalkonium chloride	100	3.12
	5	Miconazole	250	0.49
		Benzalkonium chloride	25	0.79
	1-3	Miconazole	250	0.49
		Cetrimonium chloride	25	0.78
	1	Miconazole	250	0.49
		Benzethonium chloride	100	3.1
	2-3	Miconazole	250	0.49
		Benzethonium chloride	25	0.78
	1	Miconazole	250	0.49
		Octenidine dihydrochloride	25	0.78
	2-3	Miconazole	250	0.49
		Octenidine dihydrochloride	12.5	0.39

S. epidermidis SE40	1	Miconazole	1000	2.0
		Domiphen bromide	25	0.78
	2-4	Miconazole	250	0.49
		Domiphen bromide	25	0.78
S. aureus MRSA n°34	1-4	Miconazole	250	0.49
		Domiphen bromide	100	3.1
	5	Miconazole	1000	2.0
		Domiphen bromide	100	3.1
S. epidermidis CL7	1-4	Miconazole	250	0.49
		Domiphen bromide	25	0.78
	1-3	Clotrimazole	250	0.49
		Domiphen bromide	25	0.78
	1-2	Ketoconazole	325	0.63
		Domiphen bromide	25	0.78
	3-5	Ketoconazole	250	0.49
		Domiphen bromide	25	0.78
	1-3	Voriconazole	250	0.49
		Domiphen bromide	25	0.78
		Fluconazole	1000	3.125
		Domiphen bromide	25	0.78
	1-3	Miconazole	250	0.49
		Benzalkonium chloride	25	0.78
	1-3	Miconazole	250	0.49
		Cetrimonium chloride	25	0.78
	1-3	Miconazole	250	0.49
		Benzethonium chloride	25	0.78
	1	Miconazole	250	0.49
		Octenidine dihydrochloride	25	0.78
	2-3	Miconazole	250	0.49
		Octenidine dihydrochloride	12,5	0.39
E. faecium LMG 8148	1-3	Miconazole	250	0.49
		Domiphen bromide	100	3.1
	4	Miconazole	1000	2.0
		Domiphen bromide	25	0.78
	5	Miconazole	1000	2.0
		Domiphen bromide	100	3.1
S. pyogenes ATCC 12358	1-4	Miconazole	250	0.49
		Domiphen bromide	6.3	0.20
S. dysgalactiae subsp. *equisimilis* ATCC 12449	1-4	Miconazole	250	0.49
		Domiphen bromide	6.3	0.20
B. cereus ATCC7004	1-4	Miconazole	250	0.49
		Domiphen bromide	25	0.78
L. monocytogenes ScottA	1-3	Miconazole	250	0.49
		Domiphen bromide	25	0.78
